# Recycling of Thermoset Materials and Thermoset-Based Composites: Challenge and Opportunity

**DOI:** 10.3390/polym14194153

**Published:** 2022-10-04

**Authors:** Elisabetta Morici, Nadka Tz. Dintcheva

**Affiliations:** 1 Advanced Technologies Network (ATeN) Center, Università di Palermo, Viale delle Scienze Ed. 18, 90128 Palermo, Italy; 2 Dipartimento di Ingegneria, Università di Palermo, Viale delle Scienze Ed. 6, 90128 Palermo, Italy

**Keywords:** thermoset, thermoset composites, recycling, polymer recycling

## Abstract

Thermoset materials and their composites are characterized by a long life cycle with their main applications in aircrafts, wind turbines and constructions as insulating materials. Considering the importance of recovery and valorization of these materials at their end-of-life, avoiding landfilling, the interest concerning their recycling grows continuously. The thermoset materials and their composites, to be successfully recovered and valorized, must degrade their three-dimensional structures and recover the mono-oligomers and/or fillers. The thermoset materials could successfully degrade through thermal treatment at different temperatures (for example, above 1000 °C for incineration, ca. 500 °C for oxidation/combustion of organic constituents, etc.), chemical degradation by catalyst, irradiation with or without the presence of water, alcohol, etc., and mechanical recycling, obtaining fine particles that are useful as filler and/or reinforcement additives. Among these recycling methods, this mini-review focuses on the formulation and recovery method of innovative thermoset with in-build recyclability, i.e., materials having chemical links that could be degraded on-demand or containing dynamic covalent bonds to have re-processable and/or recyclable thermoset. This issue could be considered the future perspective in developing novel thermoset materials. The aim of this review is to get an overview of the state of the art in thermoset recycling and of the most commonly used thermoset composites, recovering valuable reinforcing fibers. Additionally, in this work, we also report not only known recycling routes for thermoset and thermoset-based composites, but also new and novel formulating strategies for producing thermosets with built-in recyclability, i.e., containing chemical-triggered on-demand links. This mini-review is also a valuable guide for educational purposes for students and specialized technicians in polymer production and recycling.

## 1. Introduction

Thermosets are large molecular weight polymers that are an appealing alternative to both thermoplastics and other traditional materials, such as metals and wood, due to their structural and aesthetical advantages, cost and workability [1].

Generally speaking, thermosets are polymers cured through heat or irradiation, such as ultraviolet rays or electron beam processing, or through a chemical reaction, using a hardener or catalyst [2,3,4]. The curing process causes non-reversible chemical reactions, so the polymer chains come out crosslinked, and that is because they do not melt when exposed to high temperatures and offer superior mechanical strength. Moreover, they do not deform or lose their shape when exposed to cold temperatures. Therefore, they could be successfully used in environments in which extremely variable temperatures are recorded.

Additional advantages and enhanced properties are obtained because of the low cost of production by combining a thermoset polymer with fibers, such as carbon, glass or aramid fibers, to have thermoset composites [5,6,7]. Indeed, thermoset structural composites have been mainly used in aircraft components, on the surface and in the water transport industries, ensuring high performance for the final products and allowing savings in fuel consumption because they are lightweight [8,9]. Additionally, the high thermal, chemical and mechanical stability makes them suitable for structural and protective applications, such as aerospace materials and wind turbines [10]. It was also reported that using fiber-reinforced plastic composite materials instead of metals in airplane structures contributes to a 25% CO_2_ reduction [11].

Today, approximately 12% of the global plastic production volume, i.e., approximately 44 million tons, are thermosets; they predominantly include epoxies, polyurethanes, silicones, phenolics and also polyesters. Moreover, it is worth pointing out that the global thermosetting plastics market is projected to grow in the next few years.

In particular, epoxy resins are widely employed as engineering composites, adhesives, coatings and electrical insulation; the production of epoxy composites is evaluated to be approximately at least 4 million tons in 2030 [12].

Polyurethanes are versatile polymers with stable physicochemical properties due to their chemical composition, including urethane groups in the chains, formed by the reaction of an isocyanate with alcohol; they are mainly used in insulation foams, chemical-resistant coatings, sealants, furniture and packaging. The production of polyurethane foam is estimated to be 17 million tons in 2030 [13].

Silicones have high flexibility and resistance to heat, chemicals, sunlight and aging; typical applications include gaskets, heat insulation and soft-touch surfaces, but they are also used as lubricants and anti-foaming agents. Silicon demand is forecast to reach approximately 11 million tons in 2030 [14].

The wide and large-scale use leads to an accumulation of thermoset waste. Currently, thermoset and thermoset composite wastes are grounded up into fillers, incinerated, or digested using environmentally friendly technologies, and the vast majority are sent to landfills because they are considered difficult to recycle [15,16]. In the recent years, European legislation has required recycling of this waste instead of landfilling, but still, nowadays, no really satisfactory way has been found for thermoset composite production waste and end-of-life products. The difficulty in recycling is due to the crosslinked three-dimensional chemical nature of the thermoset matrix that cannot be re-melted by means of heat or solvent, as it happens for thermoplastic matrices, so recycling is often an expensive and low-rate process. Additionally, incineration offers poor energy efficiency and generates polluting emissions; mechanical recycling only allows for recovering lower performance reinforcements, while chemical and thermal recycling prove to be more commonly used and functional; thermoset chemical recycling consists of chemical catalyzed reactions that break down the polymer chains into building blocks and are reused in the same product as before the recycling process or in other different products [17].

In the last 20 years, the issue of recycling thermosets has been widely investigated, and although it is still a fully unsolved problem, some technologies are suitable for recycling on a large scale. The most commonly used thermosets, i.e., are polyurethane foams and epoxy composites. Therefore, polyurethane foams are usually converted into polyol, while epoxies-based composites are recycled through catalyzed alcoholysis or are converted, under certain conditions, into recyclable thermoplastics using polyamine curing agents able to cleave at crosslinking sites [18,19]. These technologies promote the circular economy and allow for business opportunities, as the waste of low-value products is turned into high-value products. Furthermore, fiber-reinforced thermosetting composites can be recycled to replace virgin materials, reducing both the raw thermoset matrix and the raw fibers used, so as to lower the environmental footprint, contributing to a more sustainable society. Researchers report that virgin carbon fiber manufacturing is a high-greenhouse-emission process. Moreover, the energy cost to produce virgin fiber is approximately 14 times higher than for the production of steel, and they should be recycled to have better resource efficiency [20,21,22].

**Figure 1:**
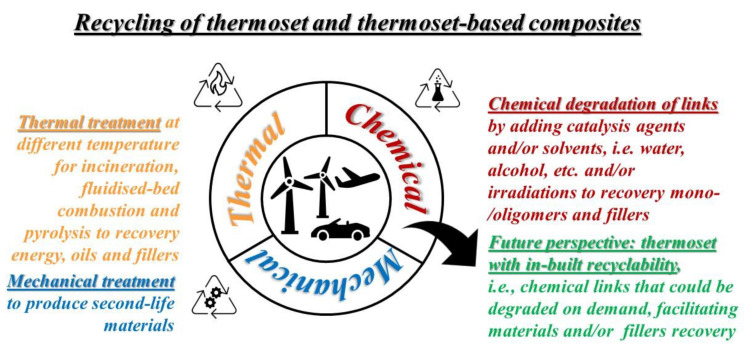
Schematic diagram to summarize current recycling strategies for thermoset and thermoset-based composites, highlighting the future perspective in developing novel thermoset materials.

Thermoset recycling has become necessary and must continue to be investigated as well to become an environmentally friendly and financially convenient solution for waste management, see Figure 1 and Table 1. The aim of this review is to get an overview of the state of the art of thermoset recycling infrastructure and of the technologies that are able to recycle the most commonly used thermoset composites, while recovering the most valuable reinforcing fibers. Additionally, in this work, we also report on the new formulating strategy to produce thermosets with built-in recyclability, i.e., containing chemical-triggered on-demand links.

## 2. Thermal Recycling

**Figure 2:**
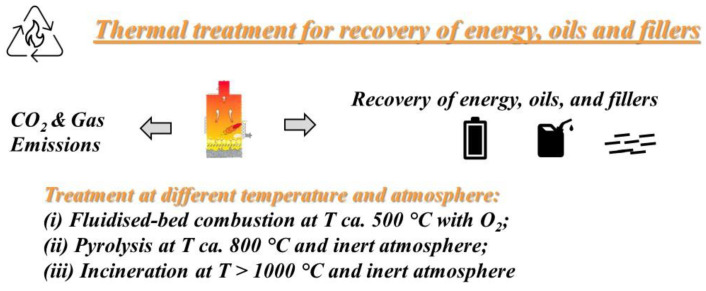
Schematic diagram to illustrate the thermal treatment and recovery of energy, oils and fillers.

The thermal recycling techniques, involving processing at high temperatures, can be mainly classified as combustion or incineration, for only energy recovery and fluidized-bed combustion to principally recover reinforcement fibers, and anaerobic combustion, i.e., pyrolysis, to recover simpler molecules used as feedstock for new chemical processing, see Figure 2.

### 2.1. Incineration

Thermosetting polymers can be burned in an incinerator for energy recovery. The advantage of this technique involves the continuous processing of waste, mixed with other types of municipal solid waste and/or contaminated waste streams. The drawbacks are connected with legislative restrictions already enforced in some European countries, with public opinion resistance about the environmental effects of the harmful generated gases, and no material recovery is achieved [23].

In some measures, economic and ecological trouble can be overcome by co-processing thermoset composite waste in cement kilns. The waste parts turn into valuable new material since the thermoset matrix burns, providing heat energy, and the incombustible residues and fibers are used as raw material for the cement clinkers. Therefore, co-processing is both material and energy recycling, which allows us to recover natural resources and save fossil fuels [24,25].

However, some additives or mineral fillers enclosed in the thermoset matrix, such as calcium carbonate and/or flame retardants, absorb energy or deteriorate during the process, lowering the amount of energy gained by incineration [26].

### 2.2. Fluidised-Bed Combustion

The process is mainly targeted at fiber recovery. The University of Nottingham was the first to develop a fluidized bed process for carbon fiber recovery. At approximately 500 °C, the organic matrix is volatilized and separated from the other components in a fluidized bed of silica sand; the air presence ensures that the polymer matrix is oxidized, so the fibers are clean. Nevertheless, a secondary combustion chamber operating at approximately 1000 °C is necessary to fully oxidize the organics. The fibers are carried out in a gas stream and then recovered in a cyclone or in a rotating separator. The organic components are used for energy and heat recovery for the process, while the fillers can be used as raw materials [27]. The drawbacks of the fluidized-bed combustion are related to the lower mechanical properties of the recycled fibers with respect to the virgin fiber, and also because of their discontinuous and flocculate nature, high temperature, pressure of the process and high energy consumption.

### 2.3. Pyrolysis

The pyrolysis process is based on heating without oxygen so that the polymeric matrix is decomposed to produce low molecular weight products in the form of liquids or gases, while the inorganic components remain unmodified. As a result, the organic part can be re-used as fuel or as new resources for chemical processes, and the fibers and the other recovered materials can be used as fillers or reinforcements for assembling the new products.

Currently, a few industrial plants around the world are already working on this. It is worth pointing out that some issues have to be solved. For example, in sheet molding compounds, the favorable temperature for the operating process is estimated to be approximately 600 °C, but undesirable degradation processes for the inorganic part could occur [28].

Furthermore, the removal of toxic compounds, such as bromine, could be necessary before pyrolysis so that the produced oils are safe to be used as fuels or as petrochemical industry feedstock [29].

Post-treatments, such as fiber sizing, could be required for re-use applications and also the quality of the recovered fiber has to be satisfied to have an effective use. A two-temperature step pyrolysis treatment for glass fiber recovery was investigated using industrial thermoset composites, i.e., wind turbine blades and automotive sheet molding. The results showed that performed fibers improved tensile strengths and failure strains but also that pyrolysis optimization can improve the quality of recovered fibers, taking care to reduce the growth rate of pre-existing surface damages during the process [30].

Microwave pyrolysis, i.e., the decomposition of organic parts of composite materials through microwave heating to have fast heating jointly to lower energy consumption, was also explored. The method has several advantages over conventional pyrolyses, such as uniform internal heating and easy control, but still, nowadays, it remains a laboratory-scale process. Technical challenges need to be overcome, such as the control of dielectric properties of the feedstock, and the optimal operating conditions still need to be investigated to prevent, for example, char residue on the fiber surface, compromising their mechanical properties [31].

## 3. Mechanical Recycling

Mechanical recycling is an extensively investigated technique since it is simple and economical. It consists of the reduction in the size of waste to re-use as powdered filler or partially reinforced fibrous materials to produce new composites, or they can be added to asphalt and cement [32,33,34].

**Figure 3:**
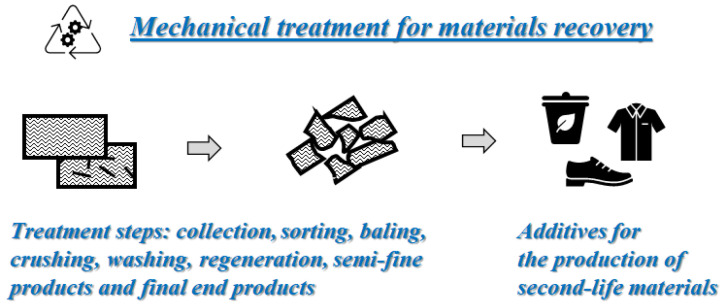
Schematic diagram to illustrate the mechanical recycling and recovery of materials.

Essentially, they consist of different steps: collection and sorting, which are challenging and require long-time processing, followed by a crushing process and further pre-treatment, reducing the scrap components into smaller and processable sized pieces, see Figure 3. Then, using a high-speed mill to grind the material, a finer product is obtained. The recycled outcome is a mixture of polymer, fibers and fillers with a size ranging from small fibrous materials up to 10 mm in length to fine powders of less than 50 microns. The fibrous product is fiber-rich and is used as reinforcement, while the powder is matrix-rich and is often used as filler. Since this technique does not produce atmospheric pollution, it offers environmental, social and economic advantages over the other recycling techniques.

At this time, mechanical recycling is a process available at the industrial scale, but recovered fillers at times are not so economically competitive with conventional fillers, such as calcium carbonates and silicates. Furthermore, because short and non-uniform fillers and fibers can be recovered, the recycled materials have low values. Therefore, the energy saved during the process could be utilized for further development on a large scale, optimizing the overall profitability of the process. Shuaib et al. studied the impact of processing rates and their granulator capacity in relation to reducing the energy demand in the recycling of thermoset-based glass fiber. Additionally, they underline that the used bottom-up approach can model the amount of energy utilization and the environmental footprint of other recycling unit processes [35].

Further improvements and more valuable recycled products could be realized by determining an efficient way to prevent the agglomeration of fibers during re-processing and improving the interface adhesion between the fiber waste and resin matrix. Recently, successful applications were presented for fused filament fabrication 3D printing, a growing sector for short-fiber composite materials; the PLA reinforced with recycled glass fibers shows modulus and tensile strength of 18% and 19%, respectively, which are higher than those of samples reinforced with virgin glass fibers. This last result was explained by considering a better inter-facial interaction between the PLA matrix and recycled glass fibers partially covered with epoxy particles [36]. Additionally, the recycled glass fiber reinforced polymer (GFRP) was used in a double-recycling route: the incorporation into a Fe-rich silicate slag, a byproduct of non-ferrous metallurgy production, allowed for a greater level of GFRP waste incorporation (20 wt%) in the in-organic matrix and flexural strength enhancement (79%) when compared with fiber-reinforced cementitious materials [37].

## 4. Chemical Recycling

**Figure 4:**
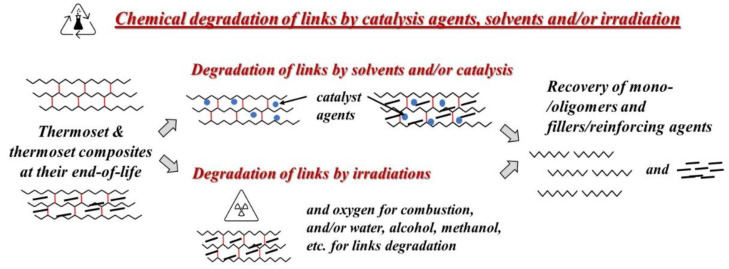
Schematic diagram to illustrate the chemical recycling and recovery of materials.

The chemical recycling of thermoset could be successfully performed considering different methods that degrade the 3D-links between the polymer chains, facilitating the recovery of mono-oligomers and fillers in the case of composites. The degradation of thermoset links could be carried out using solvents, with and without catalysis systems, and by irradiation-assisted methods, see Figure 4. Based on current understanding, the future perspective in the development of new thermoset materials will be oriented towards materials with in-build recyclability and degradability, i.e., dynamic 3D chemical links that will de-crosslink and re-crosslink “on-demand” upon external stimulus, such as pH, UV, heat and mechanical stress, see paragraph five below.

Therefore, the recovery of mono-oligomers of thermoset is a hazardous issue because of the use of solvents that could be harmful in some cases, and usually, adding catalysts is required. Although the management of thermoset waste streams must be correctly addressed, in some cases, the ecological and economic sustainability of the chemical recycling of these materials could not be a convenient issue, especially for manufacturers of second-life materials. To minimize the impact of thermoset materials and ensure correct waste management, governments must offer adequate financial support and education to people. Differently, the recovery of high-value fillers, such as carbon and aramid fibers, of thermoset composite materials could be considered a sustainable and economically advantageous issue, especially, if the fillers do not undergo any dimensional, structural and compositional changes. Obviously, using appropriate solvents (i.e., water, alcohol, methanol, ethanol, etc., also in their supercritical state) and/or catalytic agents (i.e., chemicals and/or irradiation absorbers) or irradiations (i.e., electron beam, UV irradiation, gamma irradiation, etc.), the recovery of fillers, having unchanged shape, dimensions and compositions, could be successfully and advantageously carried out.

However, as documented, the classical approach for chemically recycling thermosets and their composites is the solvolysis of crosslinked links. The main advantage of this method is the possibility of recovering unaltered fillers, although the use of solvents limits the scalability of this method at a large scale, while the solvents in their supercritical state require the use of very expensive equipment. Supercritical methanol (270 °C and 8 MPa) has been used by Okajima et al. to break the ester bonds between the epoxy backbone and to dissolve the matrix of the carbon fiber-reinforced sample. The authors demonstrated that, using supercritical fluid, the ester bonds were selectively destroyed, while both the C-C bond and the shape of the mechanical properties of the carbon fibers were preserved. This offers the opportunity to use recovered mono-oligomers and carbon fibers for the formulation of novel thermoset materials [38].

Interestingly, Liu et al. proposed the selective breaking of C-N bonds to recycle waste of epoxy-based fiber-reinforced resins coming from the aerospace industry, using ethanol as a solvent and ZnCl_2_ as a catalyst; the reaction was carried out at T = 190 °C. Based on the spectroscopy and spectrometry characterizations of productions, the authors prove that the polymer fragments have a relatively low molecular weight of 650 g/mol and terminal hydroxyl and amine groups that highlight the successful selective breaking of C-N bonds [26].

Another strategy for selectively breaking C-N bonds in amine-cured diglycidyl ether bisphenol A (DGEBA) filler reinforced resins was proposed by Wang et al. using acetic acid as a solvent and AlCl_3_ as a catalyst, and with relatively mild conditions at T = 180 °C. The spectroscopy and spectrometry analysis highlighted that the C-N bonds were replaced by N-H bonds, while the C-C and C-O bonds were preserved [39].

Upgrading to the previously commented approach, it was the introduction of an oxidizer agent that could exacerbate the cleavage of chemical bonds in the recovery of mono-oligomers from thermoset fiber-reinforced polymers, as proposed by Das et al. The authors used a mixture of acetic acid and H_2_O_2_, which resulted in the formation of peracetic acid, having string oxidation action, at relatively mild conditions, T = 65 °C and reaction time at ca. 4 h. The characterization of final products highlighted the presence of aliphatic and aromatic compounds that suggested the simultaneous and un-selective breaking of C-N, C-C and C-O bonds [40].

As discussed before, all these methods offer the possibility to recover mono-oligomers and un-altered high-value fillers, although the use of solvents, catalysts and specific conditions. Chemical recycling could be considered a valuable method for the recycling of some thermoset filler-reinforced materials, although the novel strategy related to the formulation of dynamic networks will offer further opportunities for successful recycling operations for thermoset and thermoset composites.

## 5. Thermoset with Built-in Recyclability

In the last few years, researchers have developed a new approach to overcome the difficulty of recycling thermosets by modifying the organic matrix with chemical linkers, which makes the materials much easier to break down while retaining their mechanical properties.

**Figure 5:**
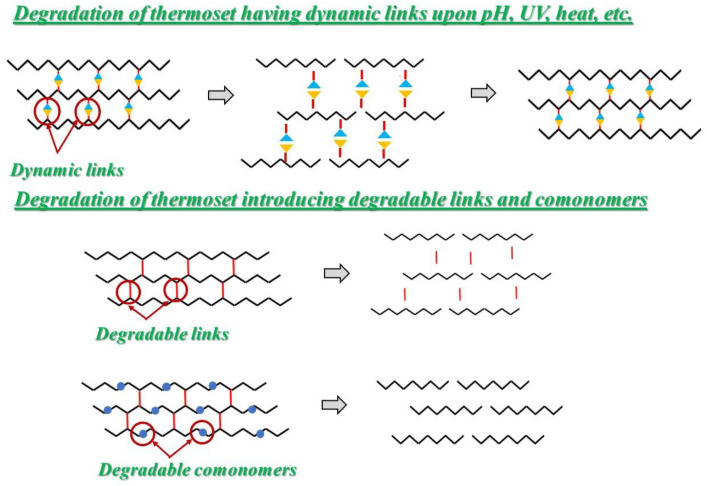
Schematic diagram to illustrate the perspective in developing the thermoset with built-in recyclability.

The idea consists of introducing degradable crosslinkers or converting permanent crosslinked structures into dynamic crosslinked ones, to have them de-crosslink and re-crosslink by means of exchange reactions of cleavable bonds, see Figure 5. These last dynamic bonds are, indeed, stimuli responsive to heat, irradiation, acid conditions and so on. They could be used to activate structural changes. Moreover, thermosets containing cleavable bonds were also used for the preparation of fiber-reinforced polymer-composites in which fibers could be easily recovered subsequent to the degradation of the resins [41,42].

While the presence of degradable crosslinkers leads to recycled polymer needing to be re-synthesized or be used in low-performing applications, the new dynamic covalent bond strategy allows direct re-shaping and recycling/re-processing, and so, conventional methods employed for thermoplastic matrices, such as injection molding or hot-press, could also be carried out for thermoset composites.

Different cleavable bonds, such as ester bonds, B–O bonds, acetal linkages, nitrogen or phosphorus-containing structures, disulfide bonds, peroxide bonds, etc., have been largely investigated for the preparation of recyclable thermosets [43,44,45,46,47].

It was established that the presence of a thermally labile tertiary ester linkage in an epoxy thermoset system lowered the decomposition temperature of the matrix; it happens that some of the degraded components form new anhydride crosslinks that could be later eliminated with proper solvents [48]. An efficient method for the chemical degradation of anhydride-cured epoxy using the phosphotungstic acid aqueous solution as the catalyst system at a mild reaction temperature of 190 °C was reported. Throughout the reaction, the ester bond in the crosslinked structure was selectively broken to obtain oligomers containing reactive groups that in turn were used for the preparation of a new anhydride-cured epoxy system [49].

Niu et al. were the first to take advantage of the B-O boronic esters bond that could undergo reversible depolymerisation via hydrolytic cleavage to prepare self-repairing polymers [50]. Degradable polyurethane thermosets having high mechanical strength and toughness were synthesized by crosslinking isocyanate-terminated prepolymers with boric acids; the presence of a labile under mild acid condition triple boron–urethane bonds, due to the reaction between hydroxyl groups in boric acids and isocyanate groups in the prepolymers, leads to a significant enhancement in the mechanical properties of the degradable polyurethanes [51].

IBM researchers well-tested cycloaliphatic diepoxides with cleavable acetal links dissolving in acid-containing solvent mixtures [52]. Acetal groups were also introduced to methacrylates to generate re-workable UV curing coatings; the acid could be produced by UV-cured methacrylates using a photoacid generator, so thermal degradation was achieved without adding additional acids [53].

The epoxy resin containing dynamic disulfide bonds was synthesized to be an excellent re-processable resin. Service temperature, thermal stability and mechanical properties were comparable to common commercial epoxy resins; better welding performance and re-processability were exhibited. Ninety percent of the tensile strength was maintained after three reprocessing cycles. Moreover, the chemical degradation in a thiol-based solvent could be performed in a closed loop to recycle fiber, resin and solvent [54]. Fiber-reinforced composites based on epoxy resin with exchangeable disulfide crosslink were easily synthetized by just substituting classical diamine hardener (DETDA) with 4-aminophenyl disulfide (AFD) as a dynamic hardener; the obtained composites showed vitrimer behavior and could be re-processed, re-paired and recycled using standard processes and equipment [55].

Several studies consider the introduction of active covalent bonds in the epoxy system triggered by external stimuli, such as heat, light or irradiation [56,57,58,59,60]; although the ideal recovery rate is 100%, the high energy employed in the process leads to poorer performance of the recovered material compared with that of the original thermosets because of degradation [61].

In order to better design a material and its properties for a required application, some researchers tested dual dynamic networks, i.e., polymeric materials combining two (or more) distinct crosslinkers in one system [62,63]. In a recent work, Liang et al., reported a feasible preparation of a dual dynamic network of waterborne polyurethane (WPU) by using 1,3-dihydroxyacetone ketotriose serving as the chain extender; a covalent adaptive network was generated by the condensation of ketohydrazine and carbonyl groups. The novel WPU exhibits effective adhesive strength to metal even after four times of deconstruction-rebonding loops [64].

Finally, a complementary approach to the design of sustainable thermosets is the introduction of a small number of cleavable co-monomers. Shieh et al. established that the silyl linkers in modified industrial polydicyclopentadiene allow for the facilitation of chemical deconstruction and are more effective with respect to the introduction of degradable crosslinkers; optimizing the cleavable bond location of a controlled thermoset degradation could be achieved and the original plastic thermoset’s material properties could be improved [65].

## 6. Conclusions

**Figure 6:**
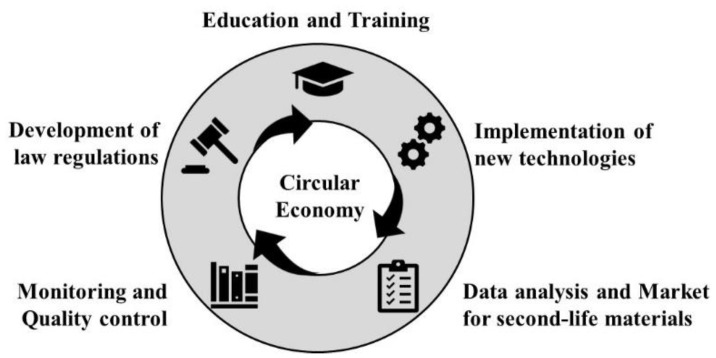
Schematic diagram to illustrate needs in transition towards circular economy.

The widespread use of thermoset composites in fields, such as aerospace, energy production and the automotive industry, coupled with landfill disposal restrictions and with the inherent value of the materials resulting from recycling, leads industries and civil societies to the need to recycle and to establish a market for recycled composite materials. Therefore, researchers must intensively investigate the recycling process, focusing on environmentally friendly and economically attractive technologies, and still today, further actions must be taken to form a growing market for recycled products. The implementation of correct recycling of thermoset and thermoset-based composites depends on numerous social, economic and cultural aspects, supporting the transition from a linear to a circular economy, see Figure 6.

Thermal recycling, indeed, must optimize the amount of energy employed in the process, and eco-friendly technologies have to be employed to lower the cost and the environmental impact. Mechanical recycling, allowing direct reuse, could be overall and successfully employed when a lower quality of the recycled material is acceptable. At least, chemical recycling, using acidic, alkaline or catalyst solutions, promotes the recovery of monomers and oligomers, which can be reused to prepare thermosets and/or their functional materials. Anyway, the use of harmful and concentrated chemicals, catalyst agents and the requirements for more steps to complete the recycling process can lead to a poor eco-friendly process that, at this time, is difficult to implement on a large scale.

To overcome the difficulty of thermoset composite recycling and the high recycling costs, a new strategy for thermosets with inherent recyclability was developed. The new topic of research was addressed to create easy matrix removal of recycling through low-energy molecular de-bonding or covalent networks, which function via either associative or dissociative mechanisms. In the last decade, the formulation of novel thermoset materials having built-in specific functions that are cleavable linkers or cleavable co-monomers triggered by external stimuli, such as thermal, chemical or optical stimuli, has been largely investigated. The potential of the new approach to stimuli-responsive materials must be fully exploited. Future perspectives are, indeed, stimulating and promising for new frontiers in material science to replace traditionally non-recyclable thermosets with more sustainable ones.

## Figures and Tables

**Table 1:** Main advantages and disadvantages of different recycling processes.

Recycling Process	(+) Advantages and (−) Disadvantages of Different Recycling Processes
Thermal recycling	(+) Recovery of energy, oils and fillers (+) No use of solvent and catalysts agents (−) Reduced dimensions and properties of recovered materials (−) Production of emissions and CO_2_
Mechanical recycling	(+) Recovery of fillers and matrix that can be used as additives to produce second-life materials (−) Long time pre-treatments to recover the materials (−) Low mechanical performance of recovered materials (−) Impossibility to re-manufacture the materials
Chemical recycling	(+) Recovery of mono-oligomers and fillers (+) Recovered fillers with unchanged shape, dimensions, composition and mechanical properties (−) Use of solvents and catalysts agents (−) Difficult to implement the process at a larger scale

## Data Availability

Not applicable.
